# Development of a novel customized cutting and rotating template for Bernese periacetabular osteotomy

**DOI:** 10.1186/s13018-019-1267-x

**Published:** 2019-07-16

**Authors:** Xuyi Wang, Shixian Liu, Jianping Peng, Zhonglian Zhu, Linlin Zhang, Jianzhong Guan, Xiaodong Chen

**Affiliations:** 1 Department of Orthopaedics, The First Affiliated Hospital of Bengbu Medical College, Bengbu, Anhui China; 2 Department of Orthopaedics, Xinhua Hospital affiliated to Shanghai Jiaotong University School of Medicine, Shanghai, China; 3 The First Affiliated Hospital of Bengbu Medical College, Bengbu, Anhui China; 4 Department of Biomedical Engineering, Shanghai University Of Technology, Shanghai, China

**Keywords:** Periacetabular osteotomy, Developmental dysplasia of the hip, 3D printing technology, Customized template

## Abstract

**Background:**

Bernese periacetabular osteotomy (PAO) has been shown to be applicable as a hip-preserving technique for the treatment of developmental dysplasia of the hip (DDH). The approach could be designed preoperatively using various types of reverse-engineering software and finite element analysis, but how to implement it in the actual PAO remains a challenge. This study examines and evaluates a solution to achieve higher accuracy when performing a PAO.

**Methods:**

A patient-specific cutting and rotating template was predesigned through computer-aided design (CAD) with three-dimensional (3D) modeling programs. The templates were then reproduced with rapid prototyping (RP) technology and used in the actual PAO. Finally, the clinical and radiographic effects were assessed and compared between the newly developed PAO and conventional PAO groups.

**Results:**

The customized cutting template fit well with the bone surface and served as a guide for surgeons as they slid the osteotome to the precise location that had been determined prior to surgery. A very similar acetabular fragment was reproduced, and no major complications occurred when performing the osteotomy along the edge of the cutting template. The acetabular fragment was then corrected to the predetermined position through one-off manipulation with the customized rotating template. The final position of the acetabular fragment in the new developed PAO group was highly consistent with the planned position, and the postoperative morphological parameters were consistent with the preoperative planned data compared to the conventional PAO group. The duration of the operation and the number of irradiation decreased significantly. The Harris hip score (HHS) and visual analogue scale (VAS) score improved significantly with the use of the new developed PAO.

**Conclusions:**

We demonstrate that our system, which was based on CAD-RP technology, is feasible and could realize the predicted results accurately during the actual PAO.

## Background

Developmental dysplasia of the hip (DDH) is a complex, three-dimensional (3D) deformity characterized by variances in the shape, size, and orientation of the acetabulum and/or proximal femur [1–3]. The prevalence of DDH varies significantly among different racial groups, from 0.1‰ in Hong Kong, China, to 75‰ in Greece and Italy [4]. Acetabular dysplasia is a primary cause of secondary osteoarthritis of the hip in adolescents and young adults [5–7]. Early and prompt surgical intervention for DDH is one of the most effective ways to prevent or delay further degeneration of the hip joint. Many surgical procedures have been reported to improve joint coverage and femoral head-acetabular congruency [8–10]. Among these, Bernese periacetabular osteotomy (PAO), proposed by Ganz et al. [11], because of its advantages such as the dorsal pillar remains mechanically intact without compromising the dimensions of the birth canal and allowing excessive potential for acetabular reorientation, has become the preferred hip-preserving surgery for treating DDH at many centers worldwide. Various studies have reported good to excellent PAO results in both clinical and radiological observations with both mid- and long-term follow-ups [12–14].

Over the last few years, with the growing use of computer technology in medical imaging, computer-aided virtual surgery based on a 3D model has been widely used in clinical settings [15, 16]. The reconstructed 3D model can be evaluated from any perspective. Surgeons can identify the location and severity of an acetabular deficiency preoperatively and apply finite element analysis to determine the optimal corrections of the acetabular fragment [17, 18]. The correction of the acetabular fragment relies largely on the surgeon’s clinical experience in conventional PAO. However, accurately implementing the preoperative planning during the actual procedure is still a challenge for surgeons. Some studies have applied computer-assisted navigation [19, 20] to improve the accuracy of surgery. However, these techniques have several disadvantages, including the need for expensive equipment, a long learning curve, a time-consuming process, and errors that occur due to shifts in the registration frame [21]. Another method for transferring the surgical plan to the operating field involves the use of various customized templates that are designed with computer-aided design (CAD) technology and manufactured with rapid prototyping (RP) technology [22, 23]. Compared with conventional surgery, the customized template has obvious advantages in simulating preoperative procedures, which are helpful in determining the feasibility of surgery and selecting the implant type, size, and position in a complex total hip replacement. CAD-RP technology has been widely used in other orthopedics procedures. However, there have been no studies on the use of this technology for PAO [24].

Based on the application of this technology in other types of surgery, we developed a computer-assisted surgical planning and 3D model-based cutting and rotating template for PAO in 2014. The aim of the present study is to describe the procedure, evaluate the feasibility and accuracy of this method, and compare our short-term results with those of conventional PAO.

## Methods

### Patients and data acquisition

The study protocol was approved by our institutional review board, and informed consent was obtained from all patients. All investigations conformed with the ethical principles of research.

From May through December 2017, we examined 20 hips in 20 consecutive subjects who had experienced hip pain for at least 6 months and were diagnosed with acetabular dysplasia by physical examinations and roentgenography. Of these, 8 hips in 8 cases (average age, 26 ± 8 years; range, 16 to 38 years) were treated with PAO using the customized cutting and rotating template, while the remaining 12 hips in 12 cases (average age, 25 ± 5 years; range, 18 to 35 years) were treated with conventional PAO. All cases selected for the study were all Crowe I or Crowe II [25] and ≤ grade 1 according to the Tönnis grading system. Each eligible patient completed a baseline evaluation comprising questions about pain severity and functional status and underwent a physical examination. The patients who had hip surgery history (such as open reduction), other hip deformities (such as Legg–Calvé–Perthes), proximal femoral deformity (such as coxa varus and/or valgus), Crowe III~IV, and Tönnis grade of 2 were excluded. Participants who were referred to the outpatient center on odd and even days were assigned to the conventional PAO or the new developed PAO group, respectively. The subjects’ clinical data are summarized in Table 1.

**Table 1:** The clinical data of the subjects included in this study

	New developed PAO	Conventional PAO	*p* value
Numbers (hips)	8 (8)	12 (12)	
Female/Male	5/3	7/5	
Ages (years)	26 ± 8 (16 to 38)	25 ± 5 (18 to 35)	0.8549
BMI (kg/m^2^)	21 ± 2 (19 to 23)	22 ± 2 (18 to 24)	0.7415
Durations (months)	13 ± 5 (6 to 20)	12 ± 5 (7 to 24)	0.07
Harris hip score	66 ± 5 (61 to 77)	67 ± 5 (61 to 78)	0.4118
Visual analogue score	3.2 ± 0.6 (2.5 to 4.5)	3.0 ± 0.4 (2.6 to 3.6)	0.1586

Values are expressed as mean ± standard deviation with range in parentheses

The preoperative and postoperative CT imaging data (from the top of the sacrum down to the level of the lesser trochanters) of each patient were obtained using the Siemens 64 channel scanner (Siemens Healthcare, Munich, Germany). Helical scanning was conducted at 120 kVp and 300 mAs, 512 × 512 matrix, 0.7539 pitch, 300 to 400 mm field of view, and 0.75 mm axial slice thickness. The patients were placed in the supine position, with their hips and knees fully extended, patellae pointing straight up, and the tiptoe stabilized at 15° of internal rotation. Care was taken to ensure that the midline of the body was aligned with the midline of the scanning table. All CT data were converted to the digital imaging and communications in medicine (DICOM) format and imported into a personal computer to generate the 3D model of the pelvis.

### Preoperative planning

**Fig. 1:**
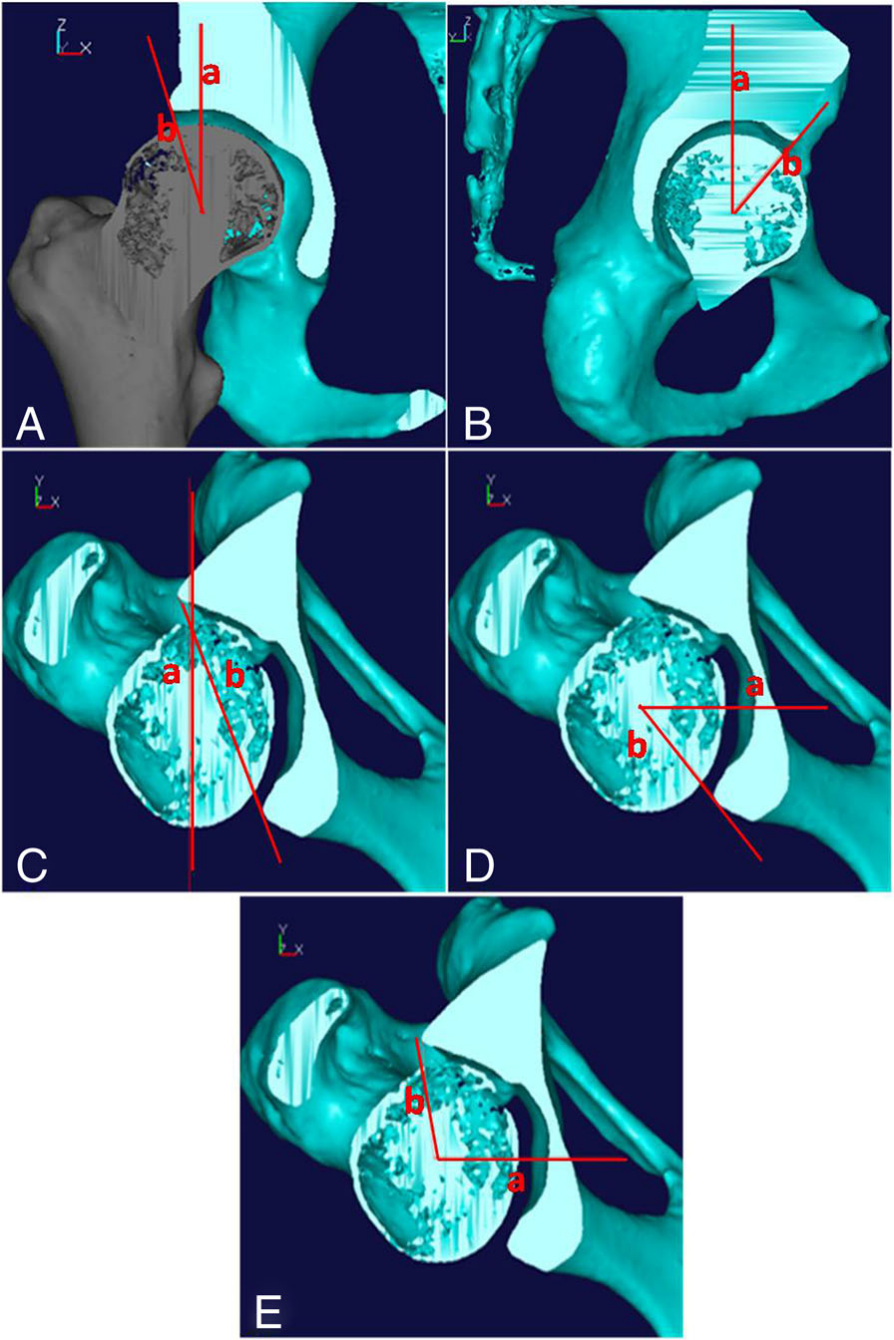
The measurement methods of acetabular angle on 3D model. **a** The coronal image passing through the center of the femoral head, the intersection angle represent LCEA. **b** The sagittal image passing through the center of the femoral head, the intersection angle represent ACEA. **c**–**e** The axial image passing through the center of the femoral head, the intersection angle represent AAVA (**c**), AASA (**d**), and PASA (**e**)

The 3D model of the pelvis was reconstructed using the Imageware 13.1 reverse-engineering software program (SensAble Technologies, Woburn, MA, USA). For each patient, the severity and direction of acetabular deficiency were evaluated by measuring the anatomical morphological data, including the lateral center-edge angle (LCEA), the anterior center-edge angle (ACEA), the acetabular anteversion angle (AAVA), the anterior acetabular sector angle (AASA), and the posterior acetabular sector angle (PASA) (Fig. 1a–e, respectively). As the position of the pelvis (i.e., pelvic tilt and pelvic rotation) has a considerable effect on the measurement of the acetabular angle, we defined the anterior pelvic plane, which involves the bilateral anterior superior iliac spines and the pubic tubercles, as the specific anatomical reference plane (the standard coronal plane) to correct the pelvic position [15]. Based on these preoperative data, we planned the rotation angle and direction of the free osteotomized fragment.

**Fig. 2:**
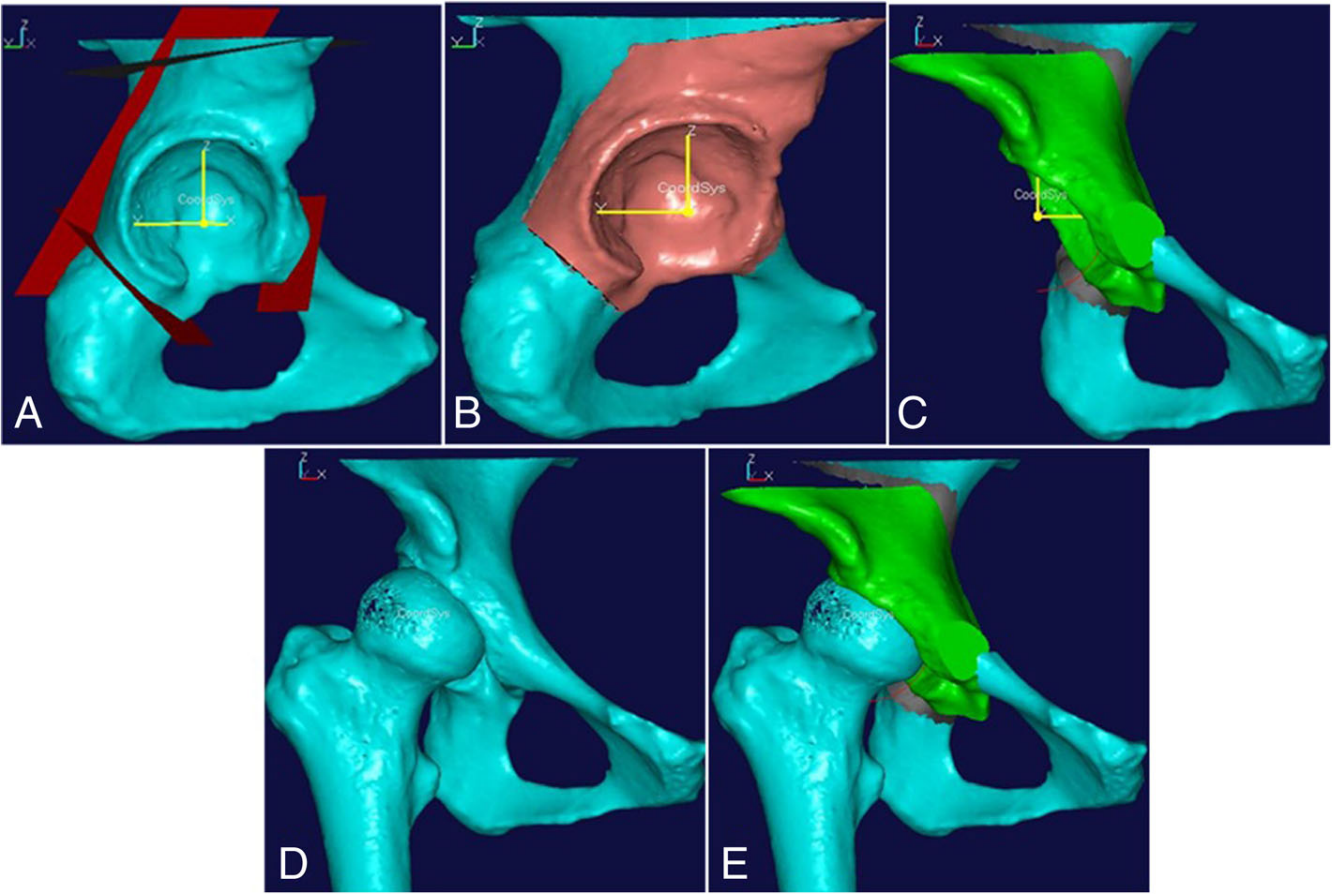
The virtual PAO was performed on the reconstructed pelvic 3D model and show the effect chart between pre and post virtual PAO. **a** The defined cutting plane according to the osteotomy method described by Ganz. **b** The free acetabular fragment after performing virtual PAO. **c** The correct position of the acetabular fragment based on the comparison between the preoperative acetabular angle and the normal data. **d**, **e** The comparison between pre and post virtual PAO shows that the femoral head coverage was improved

Based on Ganz’s description [11], we performed a virtual PAO on the reconstructed 3D model by defining the corresponding cutting plane (Fig. 2a). Cutting plane 1 was defined as a plane perpendicular to the superior ramus of the pubis and 1 cm medial to the iliopubic eminence. Cutting plane 2 was defined as a plane from the anterior of the ischial body towards the ischial spine along the infracotyloid groove. Cutting plane 3 was defined as a plane between the anterior superior iliac spine and the anterior inferior iliac spine towards the greater sciatic notch. Cutting plane 4 was at an angle of 25°–30° anteversion to the standard coronal plane with a distance of 1.0–1.5 cm from the greater sciatic notch and connecting planes 2 and 3 to form an angle of 110°–120° relative to plane 3.

An acetabular fragment almost identical to the actual PAO was reproduced through a series of periacetabular osteotomies along the margin of 4 cutting planes (Fig. 2b). Then, the rotation of the acetabular fragment was simulated anterolaterally (Fig. 2c). The degree and direction of the fragment were related to the severity of acetabular dysplasia and varied considerably among patients. After the above procedures were completed, a closing-wedge gap between the fragment and the residual pelvis was formed.

### Design and fabrication of the customized cutting and rotating template

**Fig. 3:**
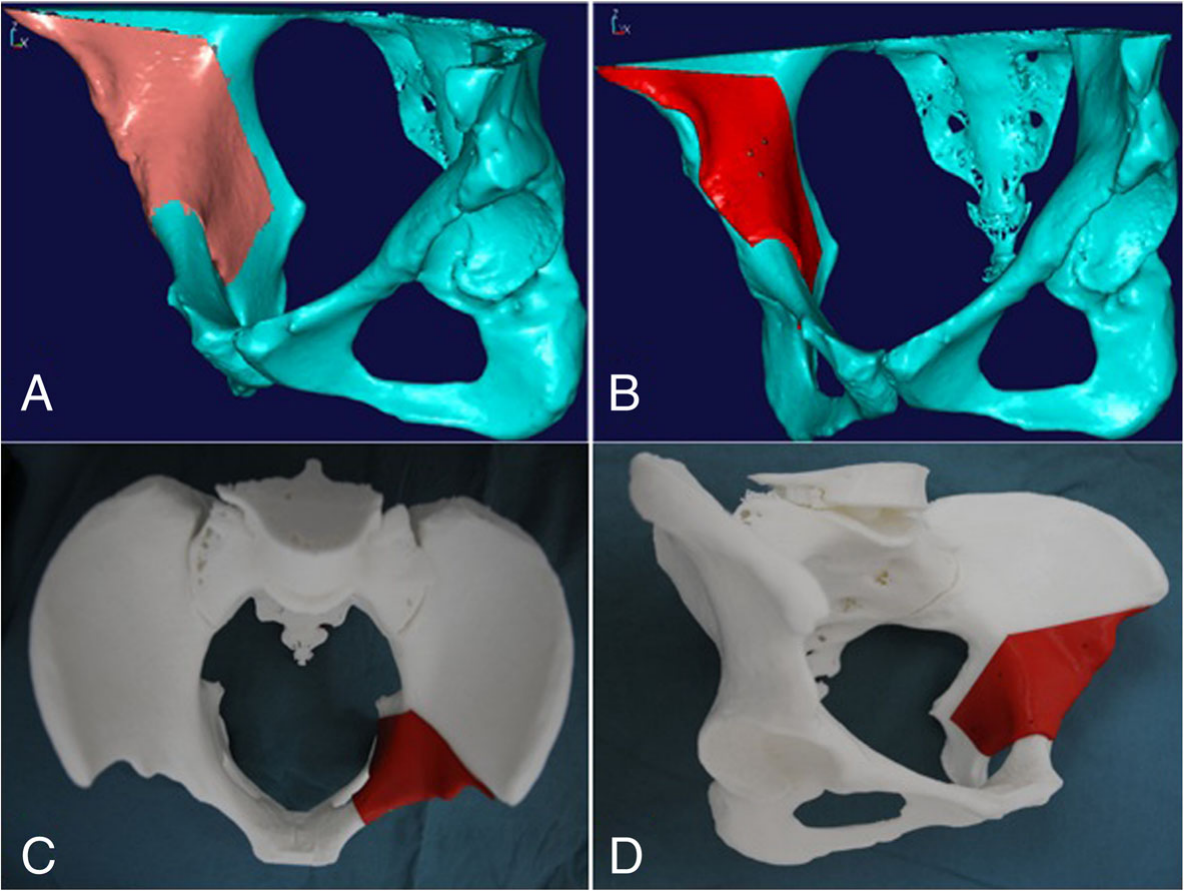
The design process and entity generation of the cutting template. **a** The preoperative planned acetabular fragment. **b** The predesigned patient-specific cutting template matched with the contour the planned acetabular fragment through computer-aided design technology. **c**, **d** The entity generation of the customized cutting template through rapid prototyping technology

**Fig. 4:**
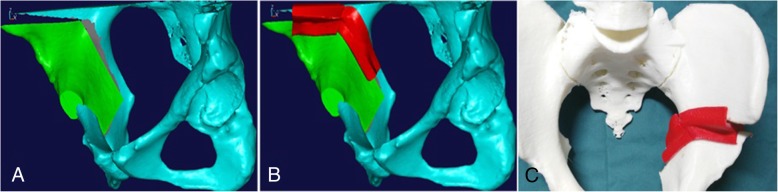
The design process and entity generation of the rotating template. **a** The preoperative planned position of the acetabular fragment. **b** The predesigned patient-specific rotating template which filled the gap between the fragment and residual pelvis through computer-aided design technology. **c** The entity generation of the customized rotating template through rapid prototyping technology

To reproduce the preoperative plan during surgery, a patient-specific cutting and rotating template was designed according to the preoperative simulation using Imageware 13.1. The details of the design process have been described elsewhere [16, 23, 26]. First, we extracted digital information regarding the free osteotomized fragment formed by the virtual PAO as described above. An appropriately sized block with two or three holes (φ2.0 mm) was placed on the surface of the acetabular quadrilateral body to be exposed during surgery. The holes were used to fix the cutting guide to the pelvis (Fig. 3a, b). Similarly, after correcting the fragment to the preoperative designed position, a unique block was reconstructed that filled the gap between the fragment and the residual pelvis (Fig. 4a, b). Second, the standard template library (STL) format data of the template were imported into FreeForm® Modeling™ version10.0 (SensAble Technologies, Woburn, MA, USA), which is another commercial 3D modeling program. After the computer-aided design of the customized template was completed, it was manufactured with medical-grade plastic materials (polyamide 12) on a plastic laser sintering machine (Formiga P100; Electro Optical Systems GmbH, Munich, Germany) (Figs. 3c, d and 4c).

### Surgical procedure

**Fig. 5:**
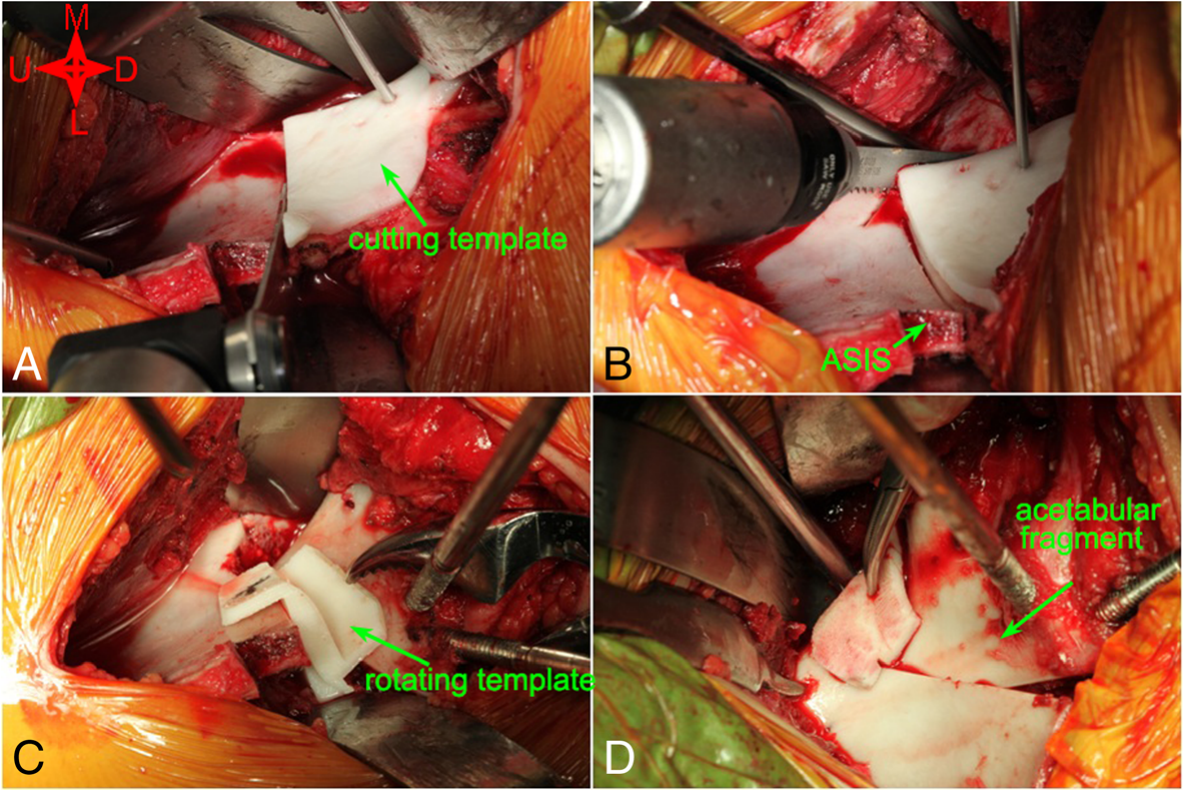
Application of the patient-specific cutting and rotating template for PAO. **a**, **b** Fixed the patient-specific cutting template on the inside surface of the pelvis and cut off the superior ramus of the pubis, ischium, ilium, and posterior acetabulum in sequence along the template edge. **c**, **d** Rotated the fragment anterolaterally and placed the rotating template into the gap between the acetabular fragment and residual pelvis and positioned the acetabular fragment at a unique location in three-dimensional space. (M: medial, L: lateral, U: up, D: down, ASIS: anterior superior iliac spine)

The surgical procedure has been described in detail by Ganz et al. [11]. A modified Smith-Peterson approach (~ 12 cm) was performed by one senior surgeon (Chen Xiaodong) in all cases. The lateral femoral cutaneous nerve was exposed bluntly in the gap between the sartorius and tensor fascia lata. The anterior superior iliac spine (ASIS) was osteotomized with the inguinal ligament; the sartorius muscle and lateral femoral cutaneous nerve were retracted medially. The inner plate of the ilium and quadrilateral body were dissected subperiosteally, and the patient-specific cutting template was placed on the inside surface of the pelvis. After confirming that the template fit closely to the bone surface and no visible gap existed, the template was fixed to the pelvis with a 2.0-mm Kirschner wire. Osteotomies of the superior ramus of the pubis, ischium, ilium, and posterior acetabulum were performed in sequence along the template edge mounted on the quadrilateral surface (Fig. 5a, b). After four periacetabular osteotomies and a controlled fracture, the completely mobile acetabular fragment was obtained. The cutting template was removed, and the fragment was rotated anterolaterally with the help of two Schanze screws. The rotating template was placed into the gap between the acetabular fragment and the pelvis and fine-tuned to ensure that the template fit properly with the acetabular fragment and pelvis (Fig. 5c, d). The reduced position of the acetabular fragment was verified by image intensifier to ensure that femoral head coverage was improved, and the motion of the hip was checked to ensure that there was no femoroacetabular impingement. The corrected acetabular fragment was fixed with three cortical screws after the rotating template was removed, and the ASIS osteotomized fragment was fixed in situ with a 3.5 mm cortical screw. The duration of operation (min), bleeding volume (ml), and irradiation times (NO) were recorded for both groups. Patients were hospitalized for an average of 8.5 days (range, 7 to 12 days).

### Postoperative assessment

One week after PAO, all subjects underwent X-ray examinations and CT scans. The acetabular angles (LCEA, ACEA, AAVA, AASA, PASA) were measured on the reconstructed 3D model of the pelvis. The accuracy of the customized template was assessed by comparing the deviation of the planned and postoperative acetabular angles in the two groups. In addition, the preoperative planned and postoperative 3D pelvic models of the new developed PAO group were superimposed to evaluate the congruence of the acetabular fragment from the geometry. The function of the hip joint was assessed using the Harris hip score (HHS). Pain of the hip joint at the time of the last follow-up was assessed with visual analogue scale (VAS) after the patient had walked for 30 to 60 min. All subjects were asked to come to our department for regular examinations after PAO at 1 month, 3 months, 6 months, and 1 year. The HHS and VAS score at the last follow-up were recorded, and the hip joint was X-rayed to evaluate recovery. Improvement at the last follow-up and the preoperative HHS and VAS score of the two groups were compared to evaluate the clinical outcomes of the new developed PAO.

### Statistics

Body mass index, duration of symptoms, and HHS and VAS score of the two groups were compared using Student’s *t* test. The duration of surgery, bleeding volume, and HHS and VAS score at the last follow-up were also compared using Student’s *t* test. Deviations of the LCEA, ACEA, AAVA, AASA, and PASA were calculated using Formula*. The deviations between the two groups were compared using Student’s *t* test. The normal distribution was estimated, and the results showed a good fit. Homogeneous variance was estimated by the *F* test, and the *F* values were greater than 0.05. Statistical analyses were conducted using SPSS (version 15.0). Significance was determined at a *p* value of < 0.05.

$$ \mathrm{Formula}\ast :\mathrm{Deviation}\ \left(\%\right)=\mid \left(\mathrm{postoperative}\ \mathrm{value}-\mathrm{planned}\ \mathrm{value}\right)/\mathrm{planned}\ \mathrm{value}\mid \times 100 $$

## Results

There were no major complications, including posterior column fracture or intraarticular osteotomy, with either surgical procedure. The average duration of the operation and the number of irradiations required in the new developed PAO group were 102 ± 7 min and 4 ± 1, while those in the conventional PAO group were 117 ± 19 min and 7 ± 2, respectively. The differences between groups were statistically significant (*p* < 0.01). The bleeding volume in the new developed PAO group was higher than that in the conventional PAO group (695 ± 119 ml vs 620 ± 45 ml), but the difference was not statistically significant (*p* = 0.0617) (Table 2).

**Table 2:** The clinical assessment of new developed PAO and conventional PAO

Parameters	New developed PAO	Conventional PAO	*p* value
Duration of operation (min)	102 ± 7 (92 to 110)	117 ± 19 (93 to 148)	< 0.01
Bleeding volume (ml)	695 ± 119 (623 to 833)	545 ± 81 (415 to 710)	0.0617
Irradiation times (NO)	4 ± 1 (3 to 6)	7 ± 2 (5 to 10)	< 0.01
Harris hip score			
Preoperative	66 ± 5 (61 to 77)	67 ± 5 (61 to 78)	0.4118
Last follow-up	94 ± 2 (92 to 97)	91 ± 1 (90 to 94)	< 0.01
Improvement	27 ± 4 (19 to 34)	24 ± 5 (12 to 30)	< 0.01
Visual analogue scale			
Preoperative	3.2 ± 0.6 (2.5 to 4.5)	3.0 ± 0.4 (2.6 to 3.6)	0.1586
Last follow-up	1.0 ± 0.8 (0 to 2.6)	1.3 ± 0.9 (0 to 2.8)	0.2531
Improvement	2.1 ± 1.0 (0.2 to 3.8)	1.7 ± 1.1 (0 to 2.9)	< 0.01

Values are expressed as mean ± standard deviation with range in parentheses

No subjects were lost to follow-up, and the mean follow-up was 12 months (range, 10 to 14 months). Preoperatively, the HHS of the two groups were 66 ± 5 (new developed PAO group) and 67 ± 5 (conventional PAO group). The VAS scores of the two groups were 3.2 ± 0.6 (new developed PAO group) and 3.0 ± 0.4 (conventional PAO group). The differences in the preoperative HHS and VAS score between the two groups were not statistically significant (*p* = 0.4118 and 0.1586, respectively). At the time of the last follow-up, the HHS had improved to 94 ± 2 in the new developed PAO group and 91 ± 1 in the conventional PAO group. The VAS score had improved to 1.0 ± 0.8 in the new developed PAO group and 1.3 ± 0.9 in the conventional PAO group. The HHS and VAS score improved significantly in the new developed PAO group compared to those in the conventional PAO group (*p* < 0.05) (Table 2).

**Fig. 6:**
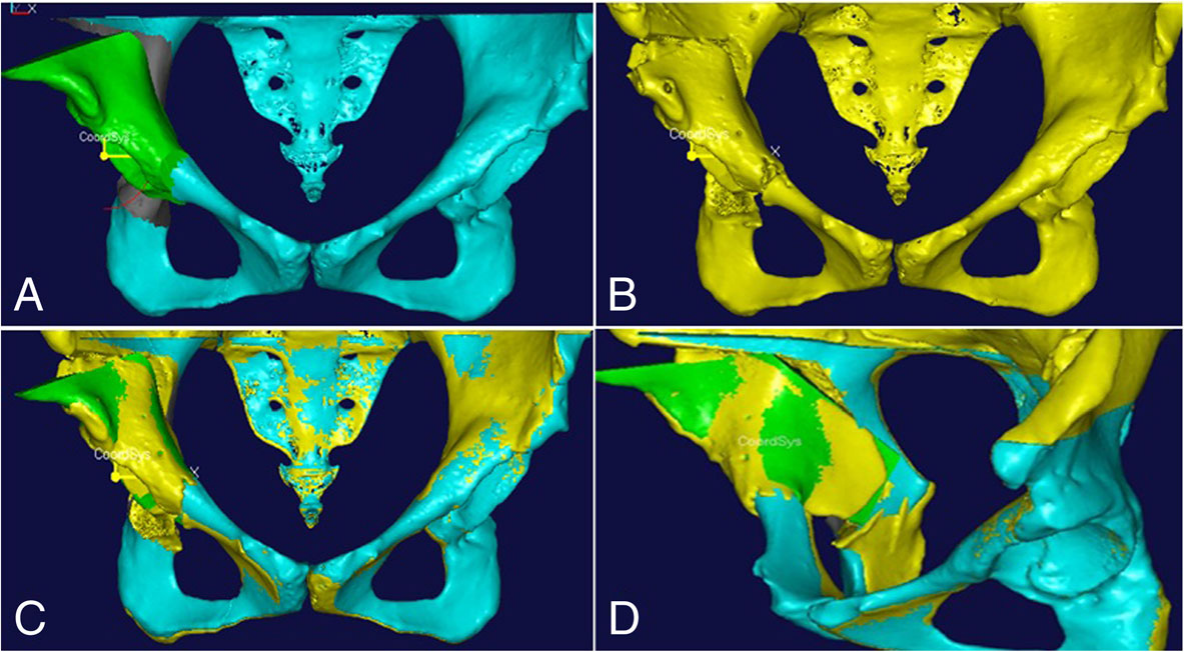
The reconstructed 3D model of the postoperative pelvis using the new developed PAO was superimposed with the one after virtual PAO. **a**, **b** The 3D model of the pelvis after virtual PAO and actual PAO. **c**, **d** The acetabular fragment produced in actual PAO was almost as same as the planned one, and the final position of the acetabular fragment was highly consistent with that planned preoperatively

Intraoperative roentgenograms and postoperative CT scanning demonstrated that femoral head coverage had improved for all subjects, and no acetabular retroversion appeared. When the acetabulum is retroverted, the anterior wall lateralizes, and a “cross-over” sign is apparent with medialization of the superoposterior acetabular wall. The customized cutting template fit perfectly with the bone surface, and there was no visible gap in any of the eight patients. An acetabular fragment almost identical in shape and size to the planned one was reproduced by performing a series of periacetabular osteotomies along the margin of the cutting template. The acetabular fragment was then corrected to its planned position with the help of the rotating template. By superimposing the postoperative and preoperative planned 3D models of the pelvis, the final position of the acetabular fragment was confirmed to be highly consistent with the planned position (Fig. 6), and the postoperative morphological parameters were quite consistent with the preoperative planned data in the new developed PAO group (Table 3).

**Table 3:** The radiographic assessment of pre and post PAO

	LCEA	ACEA	AAVA	AASA	PASA
New developed PAO					
Preoperative (°)	10 ± 5	33 ± 7	23 ± 2	42 ± 4	88 ± 3
Planned (°)	30 ± 2	53 ± 3	20 ± 1	59 ± 2	85 ± 3
Postoperative (°)	31 ± 2	54 ± 2	19 ± 1	58 ± 2	85 ± 3
Deviation (%)	4 ± 1	3 ± 3	4 ± 3	3 ± 2	1 ± 1
Conventional PAO					
Preoperative (°)	8 ± 4	32 ± 5	22 ± 2	42 ± 4	88 ± 2
Planned (°)	30 ± 2	54 ± 4	19 ± 1	58 ± 3	85 ± 2
Postoperative (°)	31 ± 3	56 ± 7	20 ± 3	59 ± 5	81 ± 3
Deviation (%)	7 ± 2	10 ± 4*	13 ± 8*	8 ± 2 *	6 ± 2*

Values are expressed as mean ± standard deviation. The deviation between planned value and postoperative value was calculated as the Formula: Deviation (%) = | (postoperative value − planned value)/planed value | × 100. *p < 0.01 compared with values in the new developed PAO group

In the new developed PAO group, the preoperative LCEA, ACEA, AAVA, AASA, and PASA were 10° ± 5°, 33° ± 7°, 23° ± 2°, 42° ± 4°, and 88° ± 3°, respectively, which were corrected to 30° ± 2°, 53° ± 3°, 20° ± 1°, 59° ± 2°, and 85° ± 3°, respectively, in the virtual PAO. The final values of these angles after the actual PAO were 31° ± 2°, 54° ± 2°, 19° ± 1°, 58° ± 2°, and 85° ± 3°, respectively. The deviations between the angles were 4° ± 1°, 3° ± 3°, 4° ± 3°, 3° ± 2°, and 1° ± 1°, respectively. In the conventional PAO group, the preoperative LCEA, ACEA, AAVA, AASA, and PASA were 8° ± 4°, 32° ± 5°, 22° ± 2°, 42° ± 4°, and 88° ± 2°, respectively, which were corrected to 30° ± 2°, 54° ± 4°, 19° ± 1°, 58° ± 3°, and 85° ± 2°, respectively, in the virtual PAO. The final values of these angles after the actual PAO were 31° ± 3°, 56° ± 7°, 20° ± 3°, 59° ± 5°, and 81° ± 3°, respectively. The deviations between the angles were 7° ± 2°, 10° ± 4°, 13° ± 8°, 8° ± 2°, and 6° ± 2°, respectively. The differences in the deviations between the new developed PAO group and the conventional PAO group, with the exception of the LCEA, were statistically significant (*p* < 0.05) (Table 3).

## Discussion

Bernese periacetabular osteotomy has been used in the treatment of residual hip dysplasia in adolescents and adults for more than 30 years [27, 28]. The amount of angular correction depends on the severity of preoperative dysplasia and varies considerably among patients. Surgeons can plan optimal, individualized operations before surgery, but how to implement it during the actual procedure remains a challenge, which is precisely the primary point for PAO [17, 18]. As insufficient correction of the acetabulum will lead to residual dysplasia, while over-correction in any dimension can cause femoroacetabular impingement [29, 30]. In addition, while PAO is a technological empirical procedure that is based on surgeon’s clinical experience, the operation itself cannot be completed under direct vision and requires a significant learning curve, which is a burden in the training of new orthopedic specialists.

In this study, we designed and fabricated a customized cutting and rotating template using 3D printing technology and applied it in the actual PAO. The customized cutting template served as a guide to help surgeons slide the osteotome into the precise location that had been determined prior to surgery without the assistance of an image intensifier during PAO. In addition, because of the specificity of the osteotomy site and direction, the possibility of serious osteotomized complications, such as posterior column splitting and/or intraarticular osteotomy, was eliminated. Obtaining the same acetabular fragment that was planned preoperatively is just the first step. Correcting the acetabular fragment to its planned position is the key to a successful PAO. The correction of the acetabular fragment in conventional PAO largely depends on the surgeon’s experience and intraoperative X-rays. However, this usually results in greater deviations due to the limitation of the incision length, the patient’s position and the surgeon’s imprecise subjective observations. Therefore, surgeons need to adjust the location of the acetabular fragment and repeatedly verify the location with an image intensifier. By contrast, the new developed PAO technique allows the fragment to be corrected to the preplanned position through one-off manipulation with the help of a customized rotating template that is embedded into the gap between the fragment and the residual pelvis. Due to its irregular contour, this customized rotating template can be used to precisely position the acetabular fragment at a unique location in the 3D space. Therefore, in our study, the final position of the acetabular fragment was highly consistent with the preoperative planned position, and the postoperative morphological parameters were consistent with the preoperative planned data in the new developed PAO group. Meanwhile, the deviations between the planned and postoperative acetabular angles in the new developed PAO group were less than those of the conventional PAO group (*p* < 0.05). These findings demonstrates that the new developed PAO technique, which uses a customized template, improves the accurate implementation of preoperative planning during the actual operation.

The postoperative record demonstrated that the new developed PAO could not only shorten the duration of the operation but also reduce the number of irradiations used, which benefits both the surgeon and the patient. Due to unskilled installation of the cutting template, the dissection scope of the soft tissue of the acetabular medial wall was larger in the first few cases, which caused the bleeding volume in the new developed PAO group to increase slightly compared with the conventional PAO group. As the techniques became more sophisticated, the bleeding volume of the new developed PAO group subjects decreased compared to the conventional PAO group. This may be because the operation was shorter and surgeons did not have to repeatedly adjust the location of the acetabular fragment with the help of customized template during the operation.

There are several limitations in the current study. First, during the preoperative planning period, we performed idealized processing by designating the femoral head center as the center of rotation and assumed the center of rotation to be fixed. However, to increase the femoral offset and abductor lever arm, the rotation center is usually transferred slightly inferiorly and medially in actual operations. The selected cases were all Crowe I or Crowe II, in which the femoral head is still located in the true acetabulum compared with subluxation or dislocation patients. Therefore, we assumed that the femoral head center could be considered the center of rotation. In addition, the displacement distance of the rotation center during the actual operation is very small relative to the femoral head radius. As a result, the impact on experimental error can be ignored. Second, the approximate cost of the customized template was RMB 1000, and several days are needed to design and fabricate the customized template. In addition, the software is relatively complicated to operate, and some training is needed to develop proficiency. Third, the greatest limitations were the small sample size and the short follow-up. Therefore, it should not jump to a conclusion that the new developed PAO using the customized cutting and rotating template has obvious advantages for the treatment of DDH compared with traditional PAO. However, we are still on the learning curve. By increasing number of patients in the study and extending the follow-up period, we believe that the efficiency of the procedure will be further demonstrated.

## Conclusions

In conclusion, this study demonstrates that our system, which is based on CAD-RP technology, is feasible and could realize the predicted results accurately during the actual PAO. The operation efficiency will further improve along with proficiency with the procedure. In the future, we plan to partner with a commercial organization to develop a more precise and convenient patient-specific osteotomy template, and we expect that it will standardize the PAO for DDH patients.

## Data Availability

All relevant raw data for this study have been presented in the main manuscript or additional supporting files, which are freely available to any scientist wishing to use them, without breaching participant confidentiality.

## Abbreviations

3D: Three-dimensional
CAD: Computer-aided design
DDH: Developmental dysplasia of the hip
HHS: Harris hip score
PAO: Bernese periacetabular osteotomy
RP: Rapid prototyping
VAS: Visual analogue scale
